# Self-reported attention and responses to cigarette package labels at the end of a two-week randomized trial of cigarette package labeling configurations

**DOI:** 10.18332/tid/189198

**Published:** 2024-06-17

**Authors:** Victoria C. Lambert, Stuart G. Ferguson, Jeff Niederdeppe, Yanwen Sun, Emily E. Hackworth, Minji Kim, Chih-Hsiang Yang, Desiree Vidaña, James W. Hardin, James F. Thrasher

**Affiliations:** 1 Department of Health Promotion, Education, and Behavior, Arnold School of Public Health, University of South Carolina, Columbia, United States; 2 College of Health and Medicine, University of Tasmania, Hobart, Australia; 3 Jeb E. Brooks School of Public Policy and Department of Communication, Cornell University, Ithaca, United States; 4 Department of Exercise Science, Arnold School of Public Health, University of South Carolina, Columbia, United States; 5 Department of Epidemiology and Biostatistics, Arnold School of Public Health, University of South Carolina, Columbia, United States

**Keywords:** smoking cessation, health warning labels, cigarette packages

## Abstract

**INTRODUCTION:**

Cigarette package inserts that describe quitting benefits and tips may promote cessation; however, research is needed to understand better their effects, including potentially enhancing the effects of pictorial health warning labels (PHWLs).

**METHODS:**

A randomized trial with a 2×2 factorial design was conducted with adult smokers (n=356) assigned to either small text-only health warning labels (HWLs; control); inserts with cessation messages, and the small text-only HWLs (inserts-only); large PHWLs (PHWLs-only); both inserts and PHWLs (inserts + PHWLs). Participants received a 14-day supply of their preferred cigarettes with packs labeled to reflect their group. Upon finishing the trial, participants reported their past 14-day frequency of noticing, reading, thinking about smoking harms and cessation benefits, talking about labels, and forgoing cigarettes because of the labels. Ordered logistic models regressed these outcomes on labeling groups, and mediation analyses assessed whether attention (i.e. noticing, reading) to labels mediated effects of labeling exposure on other outcomes (i.e. thinking about harms/benefits, talking, forgoing).

**RESULTS:**

The inserts + PHWLs group reported higher frequencies than the control group for all outcomes. Compared to the control group, both the inserts-only and PHWLs-only groups reported higher frequency of noticing (AOR=3.53 and 2.46, respectively) and reading labels (AOR=2.89 and 1.71), thinking about smoking risks because of the labels (AOR=1.93 and 1.82), and talking about labels (AOR=2.30 and 2.70). Participants in the inserts-only group also reported more frequent thinking about quitting benefits (AOR=1.98). Attention mediated all labeling effects except for the contrast between PHWLs only and control.

**CONCLUSIONS:**

Compared to text-only HWLS, cigarette labeling that involves inserts, PHWLs, or both appears more effective at drawing attention to warnings, which mediated the effects on cessation-related psychosocial and behavioral outcomes.

## INTRODUCTION

Pictorial health warning labels (PHWLs) on cigarette packages are a key policy promoted by the World Health Organization’s Framework Convention on Tobacco Control, with over 120 countries implementing them^1^. Relative to text-only warnings, PHWLs influence smoking cessation behaviors by promoting more attention toward and engagement with messages (e.g. noticing and reading), encouraging more thinking about the risks of smoking, catalyzing more conversations about warnings, and increasing motivation to quit^2-6^. PHWLs also increase rates of forgoing cigarettes that one would normally smoke, a behavior that predicts subsequent quit attempts^7,8^.

Package inserts – small-printed leaflets inside or attached to the outside of product packaging – have received much less attention from public health researchers. Canada is the only country that requires inserts for health messaging, which, in their case, describes the benefits of quitting and provides tips to quit. Observational studies^9^ and a randomized trial^10^ suggest that such inserts can promote smoking cessation behaviors. However, more research is needed to understand better the effects of these messages, including how they interact with the effects of PHWLs. The current study analyzes additional data from the aforementioned trial^10^, which was designed to assess how exposure to inserts, either alone or in combination with PHWLs, was associated with smoking-related behaviors and cognitions.

Observational, experimental, and qualitative studies have evaluated inserts with efficacy messages about cessation benefits (i.e. response efficacy) and tips to quit (i.e. self-efficacy), which is the content implemented in Canadian inserts. In Canada, post-implementation observational studies found that, compared to smokers who reported not reading inserts, smokers who read them had higher subsequent self-efficacy to quit and were more likely to both try to quit and abstain from smoking for more than 30 days^9^. Additionally, smokers previously unexposed to inserts report that efficacy messages motivate them and would help them quit^11,12^. Similarly, in a randomized case-crossover field trial, US smokers reported higher negative effects toward smoking, self-efficacy to quit, response efficacy beliefs, and motivation to quit in the week when their packs included inserts^13^. Indeed, separate analyses from this current study – a Randomized Controlled Trial (RCT) among US smokers over two weeks – found that insert exposure was associated with both more frequent thinking about the benefits of cessation and greater likelihood of forgoing or stubbing out cigarettes before they were finished^10^, both of which are consistent precursors to cessation attempts^8^.

In summary, there is strong evidence to support the use of PHWLs, and a growing body of evidence suggests that inserts with efficacy messages may also be effective. Few studies, however, have tested how these two tobacco labeling interventions interact. The potential effectiveness of combining inserts with PHWLs is supported by theories highlighting the importance of efficacy messages when messages arouse fear. For instance, many behavior-change theories posit that fear-based messages – such as those commonly used in PHWLs – are most effective when they also increase people’s confidence to engage in the recommended behavior (self-efficacy)^14,15^. Relative to text-only warnings, PHWL effects on cessation outcomes are mediated primarily by negative affect^16-18^. The ‘spotlight’ function of affect^19^ and theories of ‘emotional flow’^20^ posit that, when exposed to fear-arousing content, people search for further information about the source of their fear. As such, fear-arousing PHWLs should increase attention to insert content. Indeed, meta-analyses indicate that the inclusion of self-efficacy messages enhances the effects of fear appeals^21^.

The current study evaluates follow-up data from the RCT that found null results for labeling enhancement by combining PHWLs and inserts^10^. The analyses here extend and complement this previous evaluation by analyzing labeling effects on self-reported responses to labeling in the end-of-trial survey. Unlike the data used to test our primary outcomes, this survey asked participants to report their attention and responses to cigarette package labeling over the study period. We expect the strength of participant responses to outcomes to be strongest for the intervention groups (compared to control). Furthermore, we also expected that indicators of attention to labels (i.e. frequency of noticing and reading) would mediate any labeling effects on cessation-related responses to labeling messages (i.e. elaboration of smoking harms, elaboration of cessation benefits, talking about labels, forgoing cigarettes).

## METHODS

### Study design

**Figure 1:**
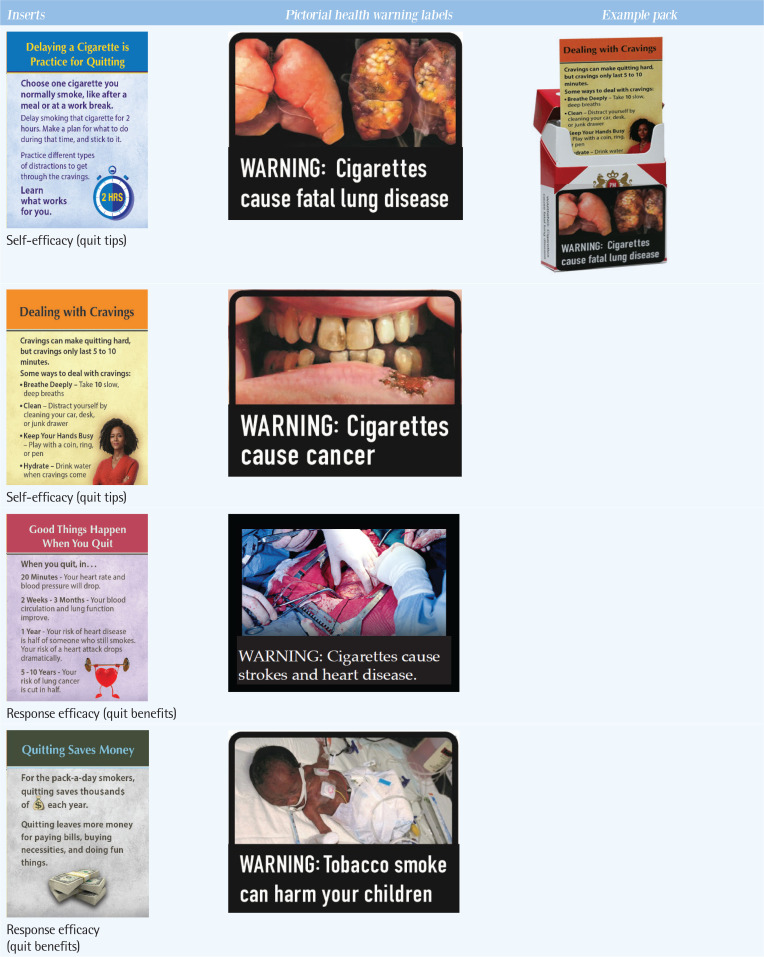
Labeling stimuli assigned to participants in a randomized controlled trial of adults who smoke from New York, South Carolina, and North Carolina, 2019-2021 (N=356)

Detailed study methods have been reported elsewhere^10,22^. Briefly, the RCT implemented a 2×2 between-subject factorial design, with participants assigned to one of four cigarette packet labeling groups: small text-only health warning labels on pack sides (control); inserts with cessation messages and the small text-only warning (inserts-only); large PHWLs (PHWLs-only); and both inserts and PHWLs (inserts + PHWLs). Packs in all groups had four different HWL texts specified for implementation in the US in 2012 (Figure 1). In the control and inserts-only groups, the current US HWL size and location were used (i.e. 50% of one pack side). In the PHWL groups – PHWLs-only, PHWLs + insert – imagery was selected based on prior research^17,23-26^, with labels affixed to the lower half of the front and back of packs. Message content for the four inserts was developed from prior studies^11,12,27^ and used low-literacy phrasing (4.6–5th grade).

On the first day of the trial, participants completed a baseline survey and received a 14-day supply of their preferred cigarette brand variety, with packs modified to reflect their experimental group. For the 14 days of the trial, participants were trained to record every cigarette they smoked using project-provided smartphones and to complete a daily evening report (see Thrasher et al.^10^ for details). On the 15th day of the study, participants completed a final end-of-study survey, which provides the data for the current study.

### Participants

Eligible adults (aged ≥18 years) had to report smoking at least 100 cigarettes in their lifetime and 10 cigarettes per day in the previous month, with exhaled CO ≥8 ppm, to verify smoking status. The latter criterion was removed after mid-way through recruitment due to the COVID-19 pandemic. People who used other nicotine products in the previous month were ineligible due to challenges with the assessment of nicotine compensation (e.g. vaping more to offset smoking less). Initial quotas for education (50% ≤ high school education) and sex (50% male, 50% female) were relaxed due to recruitment difficulties and delays caused by COVID-19. Participants were recruited in New York, South Carolina, and North Carolina using ads (e.g. flyers, social media) and, during the pre-COVID period in New York, by using intercept recruitment methods at smoke-shops in low-income neighborhoods^10,22^.

### Measurement

On the final day of the two-week study, participants self-administered a survey that included their responses to pack labeling over the study period, using or adapting questions from the International Tobacco Control Policy Evaluation Survey^28^. To assess attention, we queried how often participants reported noticing (one item) and reading or looking at (a second item) the warning labels over the prior two weeks (responses: 1 = ‘Never’ to 5 = ‘All the time’). For evaluating cognitive elaboration of labeling messages, participants reported how much the health warnings made them think about the health risks of smoking and, separately, had made them think about the benefits of quitting smoking (response options: 1 = ‘Not at all’ to 5 = ‘Extremely’). Participants also were asked about the past two-week frequency of forgoing a cigarette they were about to smoke because of the health warnings (responses: 1 = ‘Never’ to 4 = ‘Many times’), and the frequency of talking with others about the health warnings (responses: 1 = ‘Not at all’ to 5 = ‘Very often’)^29^.

### Analysis

Chi-squared tests were mostly used to assess differences across study groups, with Mann-Whitney U tests used for labeling outcomes given their non-normal distributions. Ordinal logistic models were estimated for the frequency of each of the six outcomes: noticing health warnings, reading/looking at health warnings, thinking about smoking risks, thinking about the benefits of cessation, talking about labels, and forgoing cigarettes. Indicator variables were used for each experimental group (vs the control group). For each outcome, we estimated adjusted models, controlling for baseline sociodemographics (i.e. age, race, education level, health literacy) and smoking-related variables (i.e. cigarettes smoked per day, intention to quit, recent quit attempt, self-efficacy) that predict smoking cessation attempts (Table 1). Ordinal regression models of categorical outcomes estimated coefficients that were exponentiated to allow interpretation of odds ratios. For these models, odds ratios signify the relative log odds of an outcome level or lower versus all higher outcome levels, such that odds ratios >1 indicate increased odds. All models met the proportional odds assumption.

**Table 1:** Characteristics of participants by experimental group in a randomized controlled trial of adults who smoke from New York, South Carolina, and North Carolina, 2019–2021 (N=356)

Characteristics		Control (N=98) %	Insert-only (N=83) %	PHWLs-only (N=88) %	Insert + PHWLs (N=87) %	Total sample (N=356) %
**Age** (years)	18–35	27.6	31.3	29.6	24.1	28.1
	36–55	52.0	47.0	50.5	54.0	50.8
	≥56	20.4	21.7	20.5	21.8	21.1
**Gender**	Male	39.8	39.8	42.1	31.0	38.2
	Female	60.2	60.2	58.0	69.0	61.8
**Race**	Non-White	23.5	14.3	18.9	19.5	19.2
	White	76.5	85.7	81.1	80.5	80.8
**Education level**	≤ High school	45.9	42.2	34.5	43.7	41.7
	> High school	54.1	57.8	65.5	56.3	58.3
**Health literacy^a^**	Limited	6.1	4.8	5.6	5.8	7.6
	Possibly limited	26.5	22.6	21.1	21.8	22.6
	Adequate	67.4	72.6	73.3	72.4	69.8
**Cigarettes per day**	10–15	30.6	25.3	30.7	29.9	29.2
	16–20	44.9	44.6	34.1	41.3	41.3
	>20	24.5	30.1	35.2	28.7	29.5
**Intend to quit in next 6 months**		32.7	34.9	35.2	29.9	29.8
**Quit attempt in last year**		30.6	31.3	33.0	24.1	29.8
**Recruitment period**	Pre COVID-19	42.9	46.4	50.0	42.5	45.4
	During COVID	57.1	53.4	50.0	57.5	54.6
		***Mean (SD)***	***Mean (SD)***	***Mean (SD)***	***Mean (SD)***	***Mean (SD)***
Frequency of noticing labels^b^		3.1 (1.3)	3.8 (1.3)***	3.7 (1.2)***	4.4 (0.9)***	3.7 (1.3)***
Frequency of reading labels^b^		2.8 (1.3)	3.5 (1.4)***	3.1 (1.3)	4.0 (1.1)***	3.3 (1.3)***
Thinking about smoking risks^b^		2.2 (1.2)	2.6 (1.3)	2.6 (1.2)*	3.1 (1.3)***	2.6 (1.3)***
Thinking about cessation benefits^b^		2.6 (1.4)	3.0 (1.4)	2.8 (1.4)	3.4 (1.3)***	2.9 (1.4)***
Frequency of talking about labels^b^		1.9 (1.2)	2.3 (1.2)**	2.4 (1.2)**	2.7 (1.2)***	2.3 (1.2)***
Frequency of forgoing cigarettes^b^		1.4 (0.8)	1.6 (0.9)	1.6 (0.9)	1.8 (1.0)	0.6 (0.9)*

a Measured using Newest Vital Sign^36^.

b Collected during the follow-up survey. Mann-Whitney U test (compared to control group):

* p<0.05;

** p<0.01;

*** p<0.001.

Chi-squared tests to assess differences in baseline characteristics of participants were not statistically significant.

Next, adjusted ordinal logistic regression models were used to evaluate whether attention (i.e. frequency of noticing or reading/looking at labels) mediated the effects of experimental groups on the remaining four outcomes. The two attention items were averaged (i.e. summed and then divided by two) to form a single attention measure for these models. Mediation was assessed separately for each outcome using the Karlson-Holm-Breen (KHB) method that extends the linear regression framework to the ordinal logistic regression framework^30^. The KHB method estimates mediation for categorical outcomes by separating the scaling factor from the actual effect, enabling valid comparisons of coefficients across different models while allowing decomposition of the predictor (i.e. labeling group) on the categorical outcome into both an ‘indirect effect’ that passes through and, hence, can be explained by the mediating variable (i.e. attention), as well as a ‘direct effect’ whose effect on the outcome is independent of the mediating variable. Though these coefficients could be exponentiated as in the standard ordinal logistic regression models (see above), we present unexponentiated indirect and direct effects to illustrate the direction (i.e. positive effects increase the outcome and negative effects decrease the outcome) and relative magnitude of influence. We accounted for the covariates within each mediator model as in the adjusted models. For each contrast between the experimental and control groups, the indirect effects (i.e. through the mediating variable) were estimated along with the direct effect (i.e. controlling for the mediator and adjustment variables). All statistical tests were two-tailed, with a p<0.05 significance level. All analyses were conducted using Stata v 16.1.

## RESULTS

The study sample (n=356) was mostly female and White, with about half of participants aged 36–55 years (Table 1). Most participants had more than a high school education, adequate health literacy, and did not intend to quit smoking (Table 1). Of the three intervention groups, only participants in the inserts + PHWLs group self-reported significantly more forgoing cigarettes throughout the study than those in the control group (Table 1). The self-reported frequency of noticing labels and reading label items was significantly higher in the intervention groups than in the control group (Table 1). The only exception to this finding was that participants in the PHWLs-only group had similar self-reported frequencies of reading labels compared to those in the control group. Overall means of the two attention items were similar (Table 1), and responses to the two items were strongly correlated (r=0.73, p<0.001), supporting the decision to combine the two items for subsequent mediation modeling.

Table 2 shows the results of the adjusted ordinal logistic regression models. After adjusting for covariates, participants in all three experimental groups (vs control) were more likely to notice and read labels, with the strongest effects among those in the insert + PHWLs group compared to those in the control group, participants in the inserts + PHWLs group had 6.9 times the odds of reporting a higher frequency of noticing warning labels and 5.4 times the odds of reading them (Table 2). A similar pattern of results was found for models assessing participants’ thinking about smoking risks, thinking about cessation benefits, and frequency of talking about labels; however, the contrast between the PHWLs-only group and the control group was not statistically significant in either the unadjusted (Supplementary file Table 1) or adjusted models for thinking about cessation benefits. Finally, models predicting the frequency of forgoing cigarettes indicated that only the contrast between the insert + PHWLs group and control was statistically significant, echoing differences observed by comparing mean values in Table 1. In other terms, compared to those in the control group, participants in the inserts + PHWLs group had 2.5 times the odds of reporting a higher frequency of forgoing cigarettes (Table 2).

**Table 2:** Adjusted ordinal logistic regression results for labeling group effects on self-reported attention and responses to cigarette labeling in a randomized controlled trial of adults who smoke from New York, South Carolina, and North Carolina, 2019–2021 (N=356)

*Outcome*	*Treatment group*	*AOR (95% CI)*
**Frequency of noticing**	Control ®	1
	Insert-only	3.53 (1.99–6.26)***
	PHWLs-only	2.46 (1.43–4.23)**
	Inserts + PHWLs	6.89 (3.86–12.29)***
**Frequency of reading** ®	Control ®	1
	Insert-only	2.89 (1.64–5.10)***
	PHWLs-only	1.71 (1.00–2.93)*
	Inserts + PHWLs	5.38 (3.08–9.38)***
**Frequency of thinking about smoking risks** ®	Control ®	1
	Insert-only	1.93 (1.10–3.38)*
	PHWLs-only	1.82 (1.06–3.12)*
	Inserts + PHWLs	3.36 (1.94–5.81)***
**Frequency of thinking about cessation benefits**	Control ®	1
	Insert-only	1.98 (1.14–3.44)*
	PHWLs-only	1.44 (0.84–2.48)
	Inserts + PHWLs	3.03 (1.76–5.19)***
**Frequency of talking about labels**	Control ®	1
	Insert-only	2.30 (1.33–4.25)**
	PHWLs-only	2.70 (1.53–4.77)**
	Inserts + PHWLs	3.77 (2.11–6.73)***
**Frequency of forgoing cigarettes due to labels**	Control ®	1
	Insert-only	1.63 (0.82–3.22)
	PHWLs-only	1.27 (0.63–2.56)
	Inserts + PHWLs	2.47 (1.28–4.79)**

AOR: adjusted odds ratio; adjusted by age, sex, race, education level, health literacy, cigarettes per day, intent to quit, quit attempt, and self-efficacy (all assessed at baseline). PHWL: pictorial health warning label.

* p<0.05.

** p<0.01.

*** p<0.001.

® Reference categories.

Given the observed differences between the experimental groups, we re-estimated the models to assess the contrast between the PHWL + insert group and the insert-only and PHWL-only groups, which were evaluated separately. These exploratory models are presented in Supplementary file Table 2. In most outcomes, the effect of the PHWL + insert group was significantly stronger than that observed in either the PHWL-only or inserts-only groups.

The frequency of attention to health warnings fully mediated the labeling effects found for insertsonly and inserts + PHWLs; however, none of the mediation effects (i.e. indirect effects) was statistically significant for the contrast between PHWL only and control (Table 3). The same pattern of results was found when the two attention items were analyzed separately (Supplementary file Table 3).

**Table 3:** Indirect (mediated ^a^ via self-reported attention) and direct effects of labeling group on cigarette labeling responses in a randomized controlled trial of adults who smoke from New York, South Carolina, and North Carolina, 2019–2021 (N=356)

*Outcome*	*Treatment groups*	*Mediation by Attention ^b^*	
		*Indirect effect*	*Direct effect*
		*B (95% CI)*	*B (95% CI)*
**Frequency of thinking about smoking risks**	Control ®	1	
	Insert-only	0.91 (0.15–1.67)*	-0.10 (-0.70–0.49)
	PHWLs-only	0.64 (-0.12–1.39)	0.16 (-0.41–0.72)
	Inserts + PHWLs	1.47 (0.68–2.26)***	0.12 (-0.47–0.72)
**Frequency of thinking about cessation benefits**	Control ®	1	
	Insert-only	0.69 (0.09–1.29)*	0.16 (-0.41–0.74)
	PHWLs-only	0.48 (-0.10–1.07)	-0.01 (-0.56–0.55)
	Inserts + PHWLs	1.15 (0.52–1.77)***	0.22 (-0.36–0.80)
**Frequency of talking about labels**	Control ®	1	
	Insert-only	0.71 (0.13–1.28)*	0.27 (-0.35–0.89)
	PHWLs-only	0.47 (-0.09–1.03)	0.69 (0.09–1.30)*
	Inserts + PHWLs	1.14 (0.53–1.74)***	0.48 (-0.14–1.10)
**Frequency of forgoing cigarettes due to labels**	Control ®	1	
	Insert-only	0.92 (0.18–1.66)*	-0.35 (-1.14–0.45)
	PHWLs-only	0.60 (-0.12–1.32)	-0.25 (-1.03–0.52)
	Inserts + PHWLs	1.49 (0.69–2.29)***	-0.29 (-1.05–0.48)

a All models adjusted by: age, sex, race, education level, health literacy, cigarettes per day, intent to quit, quit attempt, and self-efficacy (all collected at baseline).

b Attention calculated as the average of two attention variables: how often participants reported noticing warning labels over the prior two weeks and how often participants reported reading or looking at warning labels over the prior two weeks (Response options for both items: 1 = ‘Never’ to 5 = ‘All the time’).

* p<0.05.

** p<0.01.

*** p <0.001.

® Reference categories.

## DISCUSSION

The current study was designed to assess how exposure to inserts, both alone and in combination with PHWLs, was associated with smoking cessation-related behaviors and cognitions. We expected participants exposed to inserts – alone or combined with PHWLs – to fare better than those in the control group. Furthermore, we also expected that attention to labels would mediate these effects. The results obtained were largely consistent with these hypotheses. Our exploratory analyses suggest that the combination of inserts and PHWLs produces a greater impact on the outcomes tested than either intervention alone, consistent with theory and empirical evidence^19-21^.

With some exceptions, inserts and PHWLs produced significantly better outcomes individually than control labeling. The inserts-only and PHWLs-only groups were positively associated with more frequent talking about labels and thinking about smoking risks. These associations are consistent with other studies of PHWLs effects on elaborating risks and talking about warnings^2,3^.

Participants in the inserts-only group reported more frequent thinking about the benefits of quitting, which makes sense given that the inserted content described these benefits. That the PHWLs-only group was not significantly associated with this outcome is unsurprising, given that the depiction of consequences only implies these benefits. Indeed, a previous experimental study found that exposure to PHWLs did not affect response efficacy or perceived benefits of quitting relative to exposure to standard text-only warnings^2^. Further research is needed to determine the longer term consequences of messages that aim to promote perceived cessation benefits, including when combined with PHWL messages about smoking-related risks.

Notably, the results reported here differ from the primary analyses from this same RCT^10^: as noted in the Introduction, the primary analyses – conducted using data collected from participants in real-time during the study (i.e. Ecological Momentary Assessment around smoking sessions) – generally did not find statistically significant effects of labeling groups on psychosocial variables. Conversely, here we found that neither the inserts-only nor PHWLs-only groups were significantly associated with greater forgoing of cigarettes. Yet, the primary analyses found that these labeling groups were associated with forgoing smoking^10^. These differences could be due to methodological differences in data collection (e.g. real-time assessment vs end-of-study retrospective recall) or, perhaps more likely because our primary analyses were ultimately underpowered^10^. Study designs, like the Solomon four-group design, that systematically vary data collection approaches may be necessary to ascertain the effects of different measurement approaches.

We also found support for the hypothesis that indicators of attention to labels would mediate the effects of labeling. The frequency of noticing and reading HWLs fully mediated the labeling effects we found for inserts only or inserts + PHWLs. This is consistent with the argument that people who engage with HWLs are, in general, more likely to be influenced by them. Also, attention was significantly higher in the insert + PHWL group than in the groups exposed to either label type by itself, perhaps indicating that more information on labels promotes more message engagement. It is also noteworthy that attention did not mediate PHWL-only effects for any of the outcomes assessed. This could be due to greater avoidance of PHWLs than for inserts, perhaps due to their more aversive content and defensive response. However, some observational studies have found that avoidance of PHWLs is positively associated with subsequent quit attempts^3,31-33^ or unassociated^34^. As such, avoidance may indicate ‘ironic processing’, whereby attempts to suppress thoughts make them more likely to occur. Indeed, some research suggests that the negative effect that PHWLs generate promotes cessation behaviors directly and indirectly by promoting psychosocial and behavioral responses, including avoidance^35^. In the end, PHWLs with graphic imagery that illustrates smoking harms may promote smoking cessation through less effortful engagement than is required for processing information on cessation benefits and tips, like that we included on inserts. Further research would be necessary to confirm these speculations.

### Limitations

Our study has some limitations. Our end-of-trial measures asked participants about warning labels but did not specifically refer to inserts, as half of the participants did not receive inserts; hence, some participants may not have considered inserting messages when answering these questions. Nevertheless, the pattern of results suggests that they did. As described above, more research is needed to understand the effect of the timing of assessments and whether measures that ask directly about message responses are more or less accurate than those that do not. We changed our recruitment protocols midway through the study due to the COVID-19 pandemic (e.g. dropped smoking status confirmation through expired CO); however, as with other potential confounders, our randomized design resulted in proportional allocation of participants to labeling groups before and after the onset of COVID-19. Indeed, a major strength of our study was its experimental design and multisite recruitment, which limited potential confounding of the relationships we assessed by factors other than exposure to the messages. Our study was originally powered based on having multiple observations from each individual^10^, and the current study had lower power due to each individual contributing only one observation – that we found consistent, statistically significant effects indicates that the effects described in the current study were reasonably large. Nevertheless, studies with larger samples and longer follow-up periods are needed to evaluate better labeling effects on cessation outcomes, including the potential for differential effects across smoker subgroups (e.g. sex, nicotine dependence, quit intention). Research on this topic is also needed in other countries to determine whether regulatory context influences labeling effects, including prior PHWL implementation and other factors we did not consider.

## CONCLUSIONS

Results from this RCT contribute to the evidence that inserts, both alone and in combination with PHWLs, can be an effective tobacco control strategy. Our measures of attention to health warnings that mediated – partially or fully – the majority of the labeling effects found, is evidence that the effects seen are driven by the health warnings utilized. Researchers and policymakers should continue to explore new and novel ways to package tobacco health warnings for smokers and to use theory and research to design the optimal ways to combine these labeling strategies to boost their combined effectiveness.

## Supplementary Material



## Data Availability

The data supporting this research are available from the authors on reasonable request.

## References

[1] Canadian Cancer Society. Cigarette Package Health Warnings: International Status Report. 7th ed; 2021. Accessed May 23, 2024. https://cdn.cancer.ca/-/media/files/about-us/media-releases/2021/cigarette-health-warnings-report/ccs-international-warnings-report-2021.pdf?_gl=1*1prmnfh*_gcl_au*MTA5MjExMzY3OC4xNzE3NDg0MTg1

[2] Brewer NT, Parada H, Hall MG, Boynton MH, Noar SM, Ribisl KM. Understanding why pictorial cigarette pack warnings increase quit attempts. Ann Behav Med. 2019;53(3):232-243. doi:10.1093/abm/kay032 29850764 PMC6265120

[3] Yong HH, Borland R, Thrasher JF, et al. Mediational pathways of the impact of cigarette warning labels on quit attempts. Health Psychol. 2014;33(11):1410-1420. doi:10.1037/hea0000056 24977309 PMC4600667

[4] Boynton MH, Agans RP, Bowling JM, et al. Understanding how perceptions of tobacco constituents and the FDA relate to effective and credible tobacco risk messaging: a national phone survey of U.S. adults, 2014-2015. BMC Public Health. 2016;16:516. doi:10.1186/s12889-016-3151-5 27333921 PMC4918079

[5] Noar SM, Francis DB, Bridges C, Sontag JM, Ribisl KM, Brewer NT. The impact of strengthening cigarette pack warnings: systematic review of longitudinal observational studies. Soc Sci Med. 2016;164:118-129. doi:10.1016/j.socscimed.2016.06.011 27423739 PMC5026824

[6] Thrasher JF, Brewer NT, Niederdeppe J, et al. Advancing tobacco product warning labels research methods and theory: a summary of a Grantee Meeting held by the US National Cancer Institute. Nicotine Tob Res. 2019;21(7):855-862. doi:10.1093/ntr/nty017 29444268 PMC6775856

[7] Borland R, Yong HH, Wilson N, et al. How reactions to cigarette packet health warnings influence quitting: findings from the ITC Four-Country survey. Addiction. 2009;104(4):669-675. doi:10.1111/j.1360-0443.2009.02508.x 19215595 PMC4394051

[8] Partos TR, Borland R, Thrasher JF, et al. The predictive utility of micro indicators of concern about smoking: findings from the International Tobacco Control Four Country study. Addict Behav. 2014;39(8):1235-1242. doi:10.1016/j.addbeh.2014.04.001 24813549 PMC4043837

[9] Thrasher JF, Swayampakala K, Cummings KM, et al. Cigarette package inserts can promote efficacy beliefs and sustained smoking cessation attempts: a longitudinal assessment of an innovative policy in Canada. Prev Med. 2016;88:59-65. doi:10.1016/j.ypmed.2016.03.006 26970037 PMC4902777

[10] Thrasher JF, Ferguson SG, Hackworth EE, et al. Combining inserts with warning labels on cigarette packs to promote smoking cessation: a 2-Week randomized trial. Ann Behav Med. 2024;58(1):56-66. doi:10.1093/abm/kaad052 37738629 PMC10729784

[11] Thrasher JF, Anshari D, Lambert-Jessup V, et al. Assessing smoking cessation messages with a discrete choice experiment. Tob Regul Sci. 2018;4(2):73-87. doi:10.18001/TRS.4.2.7 PMC6395051 30828595

[12] Loud EE, Lambert VC, Porticella N, Niederdeppe J, Thrasher JF. Evaluating cigarette pack insert messages with tips to quit. Tob Regul Sci. 2021;7(3):203-209. doi:10.18001/trs.7.3.5 35546961 PMC9090199

[13] Lambert V, Ferguson SG, Niederdeppe J, Hammond D, Hardin JW, Thrasher JF. Exploring the impact of efficacy messages on cessation-related outcomes using Ecological Momentary Assessment. Tob Induc Dis. 2018;16(September):44. doi:10.18332/tid/94460 31516442 PMC6659513

[14] Bandura A. Social cognitive Tteory of mass communication. Media Psychol. 2001;3(3):265-299. doi:10.1207/S1532785XMEP0303_03

[15] Popova L. The extended parallel process model: illuminating the gaps in research. Health Educ Behav. 2012;39(4):455-473. doi:10.1177/1090198111418108 22002250

[16] Emery LF, Romer D, Sheerin KM, Jamieson KH, Peters E. Affective and cognitive mediators of the impact of cigarette warning labels. Nicotine Tob Res. 2014;16(3):263-269. doi:10.1093/ntr/ntt124 23946325 PMC3920332

[17] Evans AT, Peters E, Strasser AA, Emery LF, Sheerin KM, Romer D. Graphic warning labels elicit affective and thoughtful responses from smokers: results of a randomized clinical trial. PLoS One. 2015;10(12):e0142879. doi:10.1371/journal.pone.0142879 26672982 PMC4684406

[18] Hall MG, Sheeran P, Noar SM, et al. Negative affect, message reactance and perceived risk: how do pictorial cigarette pack warnings change quit intentions? Tob Control. 2018;27(e2):e136-e142. doi:10.1136/tobaccocontrol-2017-053972 29248897 PMC6004228

[19] Peters E, Evans AT, Hemmerich N, Berman M. Emotion in the law and the lab: the case of graphic cigarette warnings. Tob Regul Sci. 2016;2(4):404-413. doi:10.18001/TRS.2.4.10 29057296 PMC5648023

[20] Nabi RL. Emotional flow in persuasive health messages. Health Commun. 2015;30(2):114-124. doi:10.1080/10410236.2014.974129 25470436

[21] Tannenbaum MB, Hepler J, Zimmerman RS, et al. Appealing to fear: a meta-analysis of fear appeal effectiveness and theories. Psychol Bull. 2015;141(6):1178-1204. doi:10.1037/a0039729 26501228 PMC5789790

[22] Porticella N, Cannon JS, Wu CL, et al. Recruitment methods, inclusion, and successful participation in a longitudinal clinical trial using ecological momentary assessment. Health Educ Behav. 2024;51(2):280-290. doi:10.1177/10901981231210520 38008973 PMC10980577

[23] Hammond D, Reid JL, Driezen P, Boudreau C. Pictorial health warnings on cigarette packs in the United States: an experimental evaluation of the proposed FDA warnings. Nicotine Tob Res. 2013;15(1):93-102. doi:10.1093/ntr/nts094 22505660 PMC3524059

[24] Hammond D, Thrasher J, Reid JL, Driezen P, Boudreau C, Santillán EA. Perceived effectiveness of pictorial health warnings among Mexican youth and adults: a populationlevel intervention with potential to reduce tobacco-related inequities. Cancer Causes Control. 2012;23(suppl 1):57-67. doi:10.1007/s10552-012-9902-4 22362058 PMC4586036

[25] Huang LL, Thrasher JF, Reid JL, Hammond D. Predictive and external validity of a pre-market study to determine the most effective pictorial health warning label content for cigarette packages. Nicotine Tob Res. 2016;18(5):1376-1381. doi:10.1093/ntr/ntv184 26377516 PMC5942614

[26] Thrasher JF, Carpenter MJ, Andrews JO, et al. Cigarette warning label policy alternatives and smoking-related health disparities. Am J Prev Med. 2012;43(6):590-600. doi:10.1016/j.amepre.2012.08.025 23159254 PMC3504356

[27] Thrasher JF, Islam F, Davis RE, et al. Testing cessation messages for cigarette package inserts: findings from a best/worst discrete choice experiment. Int J Environ Res Public Health. 2018;15(2):282. doi:10.3390/ijerph15020282 29415523 PMC5858351

[28] Fong GT, Cummings KM, Borland R, et al. The conceptual framework of the International Tobacco Control (ITC) Policy Evaluation Project. Tob Control. 2006;15(suppl 3):iii3-iii11. doi:10.1136/tc.2005.015438 16754944 PMC2593053

[29] Thrasher JF, Abad-Vivero EN, Huang L, et al. Interpersonal communication about pictorial health warnings on cigarette packages: policy-related influences and relationships with smoking cessation attempts. Soc Sci Med. 2016;164:141-149. doi:10.1016/j.socscimed.2015.05.042 26092600 PMC4747859

[30] Smith EK, Lacy MG, Mayer A. Performance simulations for categorical mediation: analyzing khb estimates of mediation in ordinal regression models. Stata J. 2019;19(4):913-930. doi:10.1177/1536867X19893638

[31] Cho YJ, Thrasher JF, Swayampakala K, et al. Does reactance against cigarette warning labels matter? Warning label responses and downstream smoking cessation amongst adult smokers in Australia, Canada, Mexico and the United States. PLoS One. 2016;11(7):e0159245. doi:10.1371/journal.pone.0159245 27411100 PMC4943644

[32] Fathelrahman AI, Li L, Borland R, et al. Stronger pack warnings predict quitting more than weaker ones: finding from the ITC Malaysia and Thailand surveys. Tob Induc Dis. 2013;11(September):1-8. doi:10.1186/1617-9625-11-20 24330614 PMC3848583

[33] Thrasher JF, Swayampakala K, Borland R, et al. Influences of self-efficacy, response efficacy, and reactance on responses to cigarette health warnings: a longitudinal study of adult smokers in Australia and Canada. Health Commun. 2016;31(12):1517-1526. doi:10.1080/10410236.2015.1089456 27135826 PMC4972657

[34] Borland R, Yong HH, Balmford J, Fong GT, Zanna MP, Hastings G. Do risk-minimizing beliefs about smoking inhibit quitting? Findings from the International Tobacco Control (ITC) Four-Country Survey. Prev Med. 2009;49(2-3):219-223. doi:10.1016/j.ypmed.2009.06.015 19573553 PMC2766611

[35] Cho YJ, Thrasher JF, Yong HH, et al. Path analysis of warning label effects on negative emotions and quit attempts: a longitudinal study of smokers in Australia, Canada, Mexico, and the US. Soc Sci Med. 2018;197:226-234. doi:10.1016/j.socscimed.2017.10.003 29096946 PMC5758420

[36] Weiss BD, Mays MZ, Martz W, et al. Quick assessment of literacy in primary care: the newest vital sign. Ann Fam Med. 2005;3(6):514-522. doi:10.1370/afm.405 16338915 PMC1466931

