# Supplementary material for: Self-reported attention and responses to cigarette package labels at the end of a two-week randomized trial of cigarette package labeling configurations
Source: Tob Induc Dis. 2024 Jun 17;22:10.18332/tid/189198. doi: 10.18332/tid/189198 (PMC11181013; doi:10.18332/tid/189198)
Supplement: Supplementary file 1 [file TID-22-109-s1.pdf]

Supplementary Table 1. Unadjusted ordinal logistic regression model results for the effects of labeling condition on self-reported attention and responses to cigarette labeling

Outcome	Treatment group	Unadjusted Results OR(95% CI)	P-value
Frequency of Noticing	Control	Ref.	
	Insert-only	3.16(1.82-5.46)	<0.001
	PHWLs-only	2.43(1.44-4.09)	0.001
	Inserts+PHWLs	6.70 (3.80-11.80)	<0.001
Frequency of Reading	Control	Ref.	
	Insert-only	2.74 (1.59-4.71)	<0.001
	PHWLs-only	1.61 (0.96-2.69)	0.069
	Inserts+PHWLs	4.77 (2.78-8.17)	<0.001
Frequency of thinking about smoking risks	Control	Ref.	
	Insert-only	1.73 (1.01-2.98)	0.047
	PHWLs-only	1.76 (1.04-2.96)	0.034
	Inserts+PHWLs	3.30 (1.92-5.65)	<0.001
Frequency of thinking about cessation benefits	Control	Ref.	
	Insert-only	1.62 (0.95-2.75)	0.075
	PHWLs-only	1.35 (0.80-2.28)	0.260
	Inserts+PHWLs	2.64 (1.57-4.47)	<0.001
Frequency of talking about labels	Control	Ref.	
	Insert-only	2.17 (1.24-3.81)	0.007
	PHWLs-only	2.45 (1.41-4.24)	0.001
	Inserts+PHWLs	3.64 (2.07-6.43)	<0.001
Frequency of forgoing cigarettes due to labels	Control	Ref.	
	Insert-only	1.49 (0.77-2.64)	0.234
	PHWLs-only	1.37 (0.71-2.64)	0.346
	Inserts+PHWLs	2.34 (1.25-4.38)	0.008

1. adjusted by: age, sex, race, education, health literacy, number of cigarettes per day, intent to quit, quit attempt, and self-efficacy (all assessed at baseline); *: p-value of t-test (compared to control group) <0.05; **: p-value of t-test (compared to control group) <0.01; ***: p-value of t-test (compared to control group) <0.001

Supplementary Table 2. Adjusted[1] ordinal logistic regression model results for the effects of labeling condition on self-reported attention and responses to cigarette labeling

Outcome	Treatment group	Adjusted Odds Ratio (95% CI)	Adjusted Odds Ratio (95% CI)
Frequency of Noticing	Control	0.28 (0.16-0.50)***	0.41 (0.24-0.70)***
	Insert-only	Ref.	1.44 (0.81-2.55)
	PHWLs-only	0.70 (0.39-1.24)	Ref.
	Inserts+PHWLs	1.95 (1.07-3.57)*	2.80 (1.56-5.02)***
Frequency of Reading	Control	0.35 (0.20-0.61)***	0.58 (0.34-1.00)*
	Insert-only	Ref.	1.69 (0.96-2.97)
	PHWLs-only	0.59 (0.34-1.04)	Ref.
	Inserts+PHWLs	1.86 (1.05-3.31)*	3.14 (1.80-5.48)***
Frequency of thinking about smoking risks	Control	0.52 (0.29-0.90)*	0.56 (0.33-0.97)*
	Insert-only	Ref.	1.09 (0.62-1.91)
	PHWLs-only	0.92 (0.52-1.61)	Ref.
	Inserts+PHWLs	1.92 (1.10-3.36)*	2.09 (1.20-3.63)**
Frequency of thinking about cessation benefits	Control	0.54 (0.31-0.94)*	0.71 (0.41-1.22)
	Insert-only	Ref.	1.31 (0.75-2.29)
	PHWLs-only	0.76 (0.44-1.33)	Ref.
	Inserts+PHWLs	1.56 (0.91-2.70)	2.06 (1.19-3.55)*
Frequency of talking about labels	Control	0.46 (0.26-0.81)**	0.38 (0.22-0.68)***
	Insert-only	Ref.	0.84 (0.48-1.47)
	PHWLs-only	1.19 (0.68-2.08)	Ref.
	Inserts+PHWLs	1.60 (0.90-2.84)	1.34 (0.76-2.35)
Frequency of forgoing cigarettes due to labels	Control	0.65 (0.37-1.14)	0.57 (0.33-1.00)
	Insert-only	Ref.	0.88 (0.50-1.57)
	PHWLs-only	1.13 (0.64-2.02)	Ref.
	Inserts+PHWLs	1.99 (1.11-3.55)*	1.75 (0.99-3.11)

1. adjusted by: age, sex, race, education, health literacy, number of cigarettes per day, intent to quit, quit attempt, and self-efficacy (all assessed at baseline). Each column changes the reference group for the labeling contrasts, but otherwise has the same model specifications.
*: p-value <0.05; **: p-value <0.01; ***: p-value <0.001.

Supplementary Table 3. Mediation[1] (Indirect) and Direct effects of labeling treatment groups on responses to labels

Outcome	Treatment groups	Mediation by Frequency of Noticing Labels		Mediation by Frequency of Reading Labels	
		Indirect effect B (95% CI)	Direct effect B (95% CI)	Indirect effect B (95% CI)	Direct effect B (95% CI)
Frequency of thinking about smoking risks	Control	Ref.		Ref.	
	Insert-only	0.74 (0.13-1.35)*	0.05(-0.53-0.64)	0.84 (0.04-1.65)*	-0.05 (−0.64-0.54)
	PHWLs-only	0.57(-0.02-1.17)	0.17(-0.39-0.72)	0.43 (−0.37-1.22)	0.41(−0.16-0.97)
	Inserts+PHWLs	1.17 (0.54-1.81)***	0.29 (-0.30-0.87)	1.38(0.56-2.21)***	0.24 (−0.35-0.83)
Frequency of thinking about cessation benefits	Control	Ref.		Ref.	
	Insert-only	0.58(0.09-1.07)*	0.27(-0.30-0.83)	0.64(0.01-1.27)*	0.20 (−0.37-0.77)
	PHWLs-only	0.44(-0.39-0.93)	0.00 (-0.55-0.56)	0.33 (−0.29-0.95)	0.20(−0.36-0.76)
	Inserts+PHWLs	0.94 (0.42-1.46)***	0.33 (-0.25-0.90)	1.08(0.43−1.73)***	0.33(−0.24−0.91)
Frequency of talking about labels	Control	Ref.		Ref.	
	Insert-only	0.62 (0.12-1.12)*	0.34 (-0.28-0.95)	0.60(0.04−1.16)	0.38(−0.23−0.99)
	PHWLs-only	0.49 (0.01-0.98)*	0.61 (0.01-1.21)*	0.31(−0.24−0.86)	0.87(0.27−1.47)**
	Inserts+PHWLs	0.97 (0.44-1.50)***	0.55 (-0.06-1.16)	1.00(0.40−1.59)***	0.62(0.01−1.24)*
Frequency of forgoing cigarettes due to labels	Control	Ref.		Ref.	
	Insert-only	0.79 (0.16-1.42)*	-0.23 (-1.01-0.54)	0.78(0.06−1.50)	-0.25(−1.04−0.54)
	PHWLs-only	0.62 (0.01-1.23)*	-0.35 (-1.12-0.42)	0.39(−0.31−0.91)	-0.10(−0.86−0.30)
	Inserts+PHWLs	1.25 (0.56-1.95)***	-0.12 (-0.87- 0.62)	1.29(0.53−2.05)***	-0.17(−0.93−0.59)

1. All models adjusted by: age, sex, race, education, health literacy, number of cigarettes per day, intent to quit, quit attempt, and self-efficacy (all collected at baseline); *: p-value <0.05; **: p-value <0.01; ***: p-value <0.001.

©2024 Lambert V.C et al.
